# Serum Matrix Metalloproteinase Levels in Patients Exposed to Sulfur Mustard

**DOI:** 10.5812/ircmj.15129

**Published:** 2014-03-05

**Authors:** Majid Shohrati, Reza Haji Hosseini, Malek Ashtar Esfandiari, Nastaran Najafian, Bita Najafian, Abbas Golbedagh

**Affiliations:** 1 Chemical Injuries Research Center, Baqiyatallah University of Medical Sciences, Tehran, IR Iran; 2 Department of Biochemistry, Payame Noor University, Tehran, IR Iran; 3 Department of Pediatrics, Baqiyatallah University of Medical Sciences, Tehran, IR Iran

**Keywords:** Matrix Metalloproteinase, Tissue Inhibitor of Metalloproteinases, Mustard Gas

## Abstract

**Background:**

Matrix metalloproteinases (MMPs) are a group of endopeptidases which comprised of various types. These proteolytic enzymes are zinc-dependent and play role in degradation of extracellular matrix (ECM). Various types of cells such as macrophages, fibroblasts, neutrophils, synovial cells and some epithelial cells secrete MMPs. According to previous studies on bronchiolitis and respiratory tract lesions in these patients and unknown pathophysiology mechanism up to date, this cross–sectional study was performed.

**Objectives:**

The aim of this study was to compare the serum MMP level in patients with chemical injuries and normal people and also determine the role of these parameters in pulmonary disorders .

**Materials and Methods:**

In this cross–sectional study, 25 Iranian patients exposed to the sulfur mustard and 25 unexposed participants as the control group were enrolled. Serum samples were collected from two groups and stored at -70˚C until the measurement of MMPs and TIMPs. ELISA kit was used for measurement of MMP and TIMP based on the kit's instruction. For validations in measurement, all samples were analyzed duplicate and in some cases triplicate.

**Results:**

The mean level of MMP-9 in serum of chemically-injured group was 1592.42 and this amount in normal group was 679.72 .So there was a significant difference between two groups (P = 0.001) and the mean level of MMP-8 in serum of patients group was 49.10 and in normal group was 35.53. Then there was no significant difference between two groups (P = 0.197). The mean levels of MMP-1 and MMP-2 was not significantly different (P value > 0.05) in the patient and normal groups. And also the mean levels of TIMP-1 and TIMP-2 was not significantly different (P > 0.05) in the patients and normal groups.

**Conclusions:**

In summary, serum MMPs in chemically-injured has shown no significant difference with normal people except for the MMP-9.

## 1. Background

Sulfur mustard gas is a chemical substance with alkylating feature which has been used in different warfare such the war between Iran and Iraq from 1983 to 1988 and the world war I (1). Both the civilians and military force in Iran were extremely exposed to this gas during the Iraq-Iran war (2). Today more than 40000 people are suffering from the effects of this gas in Iran. Mustard gas can cause damage in different organs such as eyes, skin and lung. Pulmonary lesions are one of the chronic disorders which observed in these people (3, 4). Victims of mustard gas are extensively disabled and died because of respiratory lesions (5). Exposure to the mustard gas can cause pulmonary lesions and it is determined that more than half of these injuries involved these kinds of disorders (6). Different lung disorders can be caused by sulfur mustard. It may damage the respiratory system after exposure but late effects are more unknown. Many of patients exposed to this gas develop chronic lung disorders years after exposure (7). The exact damage mechanism of mustard gas is unknown (8). We have previously carried out some studies about the pathophysiology of lung disease in these patients such as antioxidants and α-1-antitrypsin (3, 9).

Since the previous studies showed that matrix metalloproteinase (MMPs) may be involved in pathogenesis of pulmonary disorders (10). In this study we are going to investigate the relation between the serum MMPs and lung disorders in people who were exposed to the mustard gas. MMPs have various types and each type belongs to a specific enzymatic family (11). They are zinc-dependent proteolytic enzymes and play roles in degradation of extracellular matrix (ECM) (12).

Various types of cells such as macrophages, fibroblasts, neutrophils, synovial cells and some epithelial cells secrete MMPs (13). Secretion of MMPs can be controlled in several levels. The most important inhibitor factors of these components are TGFβ and TIMP (14, 15). Twenty-three types of MMPs are identified in human, which classified into six groups. Two main groups are: Collagenases and Gelatinases. The collagenases include MMP-1, MMP-8 and MMP-13 and the Gelatinazes include MMP-2 ( Gelatinize A) and MMP-9 (Gelatinize B) (16). MMP-9 is more related to TIMP-1 and MMP-2 is inhibited by TIMP-2 (17-19). MMPs are involved in lots of physiological and pathological disorders. In recent years it is clarified that measuring the blood MMPs levels will help to diagnosis the lung lesions (20).

## 2. Objectives

In this study we aimed to compare the range of serum MMPs including MMP-1, 2, 8, 9 and TIMP-1, 2 in chemically- injured patients with pulmonary lesions, who were exposed to mustard gas and the normal people.

## 3. Materials and Methods

This cross-sectional study was conducted using convenient sampling method on 25 patients exposed to sulfur mustard gas and 25 healthy participants as a control group at Chemical Injuries Research Center, Tehran city (Iran), in 2012. Student t-test was used for independent sampling, and the total sample size was estimated to 25 participants in each group. The estimated sample size was estimated by considering the power of 80% (1-β) and significance level of 5% and effect size = 0.8 and using GPOWER version 3.1.2.

Chemically injured people, who had exposed to mustard gas during Iraq-Iran war, had pulmonary disorders such as bronchiolitis while normal people had no background of exposure to mustard gas. We tried to collect these people in both groups with the same grade of pulmonary lesions and demographic properties such as age and gender. Inclusion criteria include that patients should have some specific conditions such proved exposure to mustard gas, bronchiolitis or chronic bronchitis proved by HRCT. The exclusion criteria were: unwillingness to continue participating in the research project, failure to complete the project and other chronic pulmonary diseases, co-infectious diseases like cell, immune diseases and addiction to cigarette or other drugs.

Ethics committee approval was obtained from ethic committee of BUMS (Baqiyatallah University of Medical Sciences, Meeting No. 13). The Participants were informed about the aim of the study and signed a written informed consent for the taped recorded interview. They were assured that these data will be kept confidential and also were informed that they could withdraw from the study. Both patient and control groups were checked up by 1 pulmonologist and their clinical and spirometry data and their demographic variables were recorded and they were entered into the study after describing the steps, reasons of the work and its eventual risks.

Ten milliliters venous blood was taken from all the patients. All samples were centrifuged by refrigerator centrifuge in 4ºC at 800 rpm for 5 minutes. Samples were taken to Chemical Injuries Research Center of Baqiyatallah Hospital and kept at -80ºC. After collecting all samples, ELISA kit from Ray Biotech Company (Germany), was used for qualitative measurement of MMP and TIMP based according to the manufacture's instruction. For confirmations, all samples were analyzed twice and in some cases three times. Before ELISA test, all samples were diluted in an appropriate amount. To measure the MMP-2 level, the serum sample did not need diluting. The samples were measured by ELISA reader at 450 nm. Data were analyzed with SPSS version 17 (SPSS Inc., Chicago, Ill., USA). Continuous variables are presented as the mean ± standard deviation (SD) and median, inter quartile range (IQR), whereas categorical data are presented as frequency and percentages. Independent t-tests or Mann-Whitney test were used for continuous variables. In this study, the probability value of 0.05 or less (P ≤ 0.05) was considered significant.

## 4. Results

The present sample comprised of 50 (100%) male with the mean age of 47.28 (SD = 9.19) years. The mean level of MMP-9 in serum of chemical injuries group was 1592.42 and this amount in normal group was 679.72. So there was a significant difference between two groups (P = 0.001), the mean level of MMP-8 in serum of patients group was 49.10 and in normal group was 35.53. Then there was no significant difference between two groups (P = 0.197). The mean levels of MMP-1 and MMP-2 were not significantly different (P > 0.05) in both groups. And also the mean level of TIMP-1 and TIMP-2 were not significantly different (P > 0.05) (Table 1). 

**Table 1.** The Mean Level of MMP-1, 2, 8, 9 and TIMP-1, 2 in Patients and Normal Groups ^a, b^

Variable	Dry Skin Patients Who Were Exposed to Sulfur Mustard			Normal			P Value
	Mean ± SD	Median	IQR	Mean ± SD	Median	IQR	
**MMP-1**	43.76 ± 24.65	39.00	29.26	41.29 ± 23.39	36.40	24.87	0.655
**MMP-2**	3.23 ± 1.45	2.82	1.96	3.97 ± 3.85	2.64	1.45	0.676
**MMP-8**	49.10 ± 37.12	39.90	41.1	35.54 ± 18.24	31.00	10	0.197
**MMP-9**	1592.42 ± 944.85	1651.80	1521.69	679.72 ± 511.73	513.05	697.28	< 0.001
**TIMP-1**	490.62 ± 248.36	406.84	259.86	557.80 ± 236.86	495.74	282.9	0.218
**TIMP-2**	95.90 ± 73.33	69.45	68.14	109.03 ± 65.09	81.97	78.48	0.130

^a^ Abbreviations: IQR, inter quartile range, MMP, matrix metalloproteinase; SD, standard deviation; TIMP, tissue inhibitor of matrix metalloproteinase.

^b^ P value reported base on Mann-Whitney test.

## 5. Discussion

The exact damage mechanism of mustard gas is unknown. Sulfur mustard interacts with alkylating proteins and nucleic acids, degradation of the cell structure and DNA damage which is its most important effect. Sulfur mustard causes local toxic effects on skin, eye and lung (8). The investigations on several lung disorders such as cystic fibrosis, asthma and COPD were done. Almost the whole structure of extracellular matrixes is degraded by MMPs. The expression of specific serum MMPs increases in broncho alveolar lavage and sputum of the people with cystic fibroses (CF) which is in relation with lung dysfunction (10).

MMPs are a group of endopeptidases which have a Zn^2+^ in their active site that functions with three histidines, that plays a catalytic role in association with a glutamic acid as a generic base. MMPs have various compositions in their active site, which play an important role in recognizing the substrate. This trait is very different in MMPs due to the cells which they are expressed and the cellular structure (21). The main places of the MMPs are in inflammated and remodeled tissues (10). These kinds of tissues are almost the main features of airway tissues in asthma. MMP-9 has a significant role in this kind of lung disorder and its exacerbation (22).

By considering different studies on asthma, COPD, bronchiolitis and respiratory tract lesions, and lack of knowledge about the exact damage mechanism of mustard gas until now, this study was performed with the aim of comparing the serum MMP levels in two different groups: chemically injured and normal people. Hoshino proved that connective tissues such as endothelial cells, fibroblasts and inflammatory cells can synthesize and secrete MMP-9. Patients with asthma have increased MMP-9 levels in submucosal and epithelial areas (23). Our results also showed an increasedMMP-9 level in the serum of chemically injured patients with pulmonary lesions compared with normal ones.

Fibril-forming collagen degradation is done by MMP-1. Naturally the secretion of this MMP is very low but it increases if the alveolar epithelial cells get reactive (24). In another study in 2001 by Imai et al. it was shown that alveolar type II cells produce MMP-1 and in emphysema patients it is over-expressed. They suggested that MMP-1 may have an important role in lung destruction in chronic respiratory disorders (25). They hypothesized that remodeling and changes in lung alveolar epithelial cells could lead to the expression of MMP-1 in the lung parenchyma (25). In 2013, Kiani et al. have shown that the serum MMP-1 level is directly related to the severity of pulmonary disorder in patients who were exposed to sulfur mustard (26). Our results showed that serum MMP-1 level increases in patient group who were exposed to sulfur mustard, but its difference was not statistically significant.

In 2007, La Rocca et al. showed that the activity of extracellular MMP-2 in human lung fibroblasts is inhibited in people who are in exposure of cigarette smoke (27). In 2013, Kiani et al. reported that MMP-2 level decreased in patients with pulmonary disorder who exposed to sulfur mustard (26). We found out in our study that MMP-2 level in serum was reduced in patient group, but the difference between patient and normal group was not statistically significant. Also Kiani et al. in 2013 proved that serum MMP-8 level did not significantly differ in patients with a history of exposure to sulfur mustard compared to normal people (26). They also showed that TIMP-1 and TIMP-2 levels do not have difference in patients with lung disorders who were exposed to sulfur mustard in comparison to the normal people (26). Our results showed that serum MMP-8 increased in patients who were exposed to sulfur mustard in comparison to the normal group. And the data for TIMP-1 and TIMP-2 in our study confirmed the same findings.

The prominent feature of the present study was the possibility of such studies merely in Iran because of the presence of veterans from the previous war. But the shortages may derive from the limited sample size. This research study shows that serum MMP-1, MMP-2 and MMP-8 and also TIMP-1 and TIMP-2 levels of chemically-injured patients have no significant changes compared to the normal people. But there is significant difference in MMP-9 between the two groups.
